# Quality control of *Lycium chinense* and *Lycium barbarum cortex* (*Digupi*) by HPLC using kukoamines as markers

**DOI:** 10.1186/s13020-016-0121-x

**Published:** 2017-01-09

**Authors:** Yuan-Yuan Li, Rui Di, Wing-Leung Hsu, Ye-Qing Huang, Hon-Yeung Cheung

**Affiliations:** 1 Research Group for Bioactive Products, Department of Biomedical Sciences, City University of Hong Kong, Hong Kong SAR, China; 2 Key Laboratory of Biochip Technology, Shenzhen Biotech and Health Centre, City University of Hong Kong, Shenzhen, 518057 China; 3 System and Translational Science Center, RTI International, Research Triangle Park, NC 27709 USA

## Abstract

**Background:**

Lycii Cortex (LyC), composed of *Lycium chinense* and *Lycium barbarum cortex* and having the Chinese name Digupi, is used to treat chronic diseases like cough, hypertension, and diabetes in Eastern Asia. However, chromatographic methods, such as TLC and HPLC, to determine the phytochemical composition of LyC have not been included in any official compendiums. This study aims to establish a validated HPLC method for quality control of LyC.

**Methods:**

Kukoamines A and B (KA and KB, respectively) were selected as markers for the HPLC method. An acetic acid solution was adopted for sample extraction because it facilitated the release of kukoamines and effectively prevented their degradation. Optimal separation of the kukoamine isomers was achieved on hydrophilic ligand-coated C18 columns with a gradient elution of acetonitrile and 0.1% (v/v) trifluoroacetic acid. The average contents and proposed contents for LyC were calculated with a *t* test and an uncertainty test based on 16 batches of authentic samples.

**Results:**

The method was validated with linearity (r^2^ = 0.9999 for both KA and KB), precision (RSD = 1.29% for KA and 0.57% for KB), repeatability (RSD = 1.81% for KA and 0.92% for KB), and accuracy (recovery of 90.03–102.30% for KA, and 98.49–101.67% for KB), indicating that the method could offer reliable results for quality control analysis of LyC. At the 95% confidence level, the calculated content limits were 1.45 mg/g for KA and 4.72 mg/g for KB.

**Conclusion:**

Compared with conventional morphological identification, the HPLC method involving KA and KB contents offers precise, objective, and quantitative results for quality control of LyC.

**Electronic supplementary material:**

The online version of this article (doi:10.1186/s13020-016-0121-x) contains supplementary material, which is available to authorized users.

## Background

Lycii Cortex (LyC) is used to treat chronic diseases like cough, hypertension, and diabetes in Eastern Asia [1–3]. Although pharmacopeias from China, Japan, and Korea have officially stipulated the source of LyC as the root bark of *Lycium chinense* and *Lycium barbarum* [4–6], the herb is often misused or adulterated with other cortexes having similar appearances. Contemporary authentication of LyC relies on morphological identification [4–6]. In contrast, inspections based on characteristic constituents are practical and objective, because monitoring of phytochemical markers is normally not only genus-specific, but also herbal function-related [7–9].

Chromatographic techniques are commonly used for both qualitative and quantitative purposes with reliable results [10]. However, a genuine chromatographic method for LyC involving unique markers is still missing in official compendiums. Although some phytochemicals such as phenolic acids and flavonoids have been recommended as markers for quality assessment for LyC [11–13], they are neither unique nor abundant in this herb. Hence, they should not be adopted.

Recently, kukoamines A and B (KA and KB, respectively) were recommended by us as unique markers for LyC [14], because they are truly bioactivity-related, genus-specific, and content-abundant in this herb. Although the LC–MS/MS method is considered to provide simultaneous and precise determination of numerous phytochemical constituents [14, 15], the high cost of the equipment and its operation has made this method less practical for daily quality control inspections.

This study aims to provide a validated method for quality control of LyC with reference to KA and KB. Based on the developed method, multiple batches of LyC were investigated and the content limits were statistically calculated.

## Methods

### Reagents

**Fig. 1:**
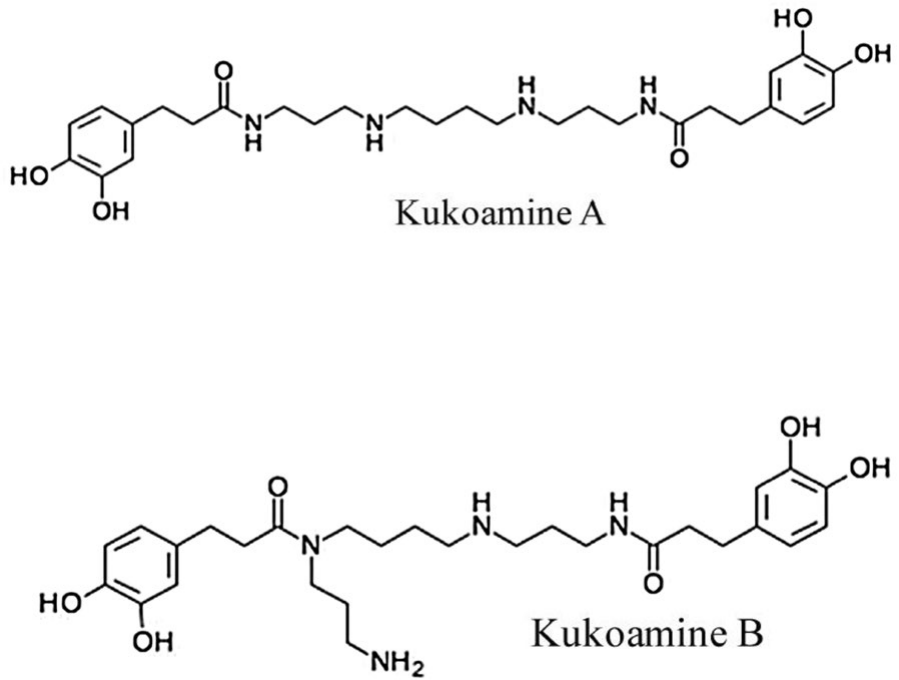
Structures of kukoamines A and B

Methanol and acetonitrile (HPLC grade) were purchased from Labscan Asia (Thailand). Trifluoroacetic acid (TFA) and acetic acid (both HPLC grade) were purchased from Fluka (Switzerland). All other chemicals were of analytical grade. Water was prepared using a Millipore MilliQ-Plus system (Millipore, USA). KA and KB (Fig. 1) were extracted and purified by our group (purity: higher than 98%) [16].

### Plant materials

Dried root bark samples of *L. chinense* and *L. barbarum* were collected from different regions in China and a local market in Hong Kong (Table 1). At least 2 kg of each bark was collected. The species of the plants were identified by Dr. Zhi-Feng Zhang (Ethnic Pharmaceutical Institute, Southwest University for Nationalities). The voucher specimens (DGP-JXXS-001, DGP-HBQL-001, and DGP-JXJJ-001) were stored in the Department of Health, Hong Kong Government SAR.

**Table 1:** Production areas and species of Lycii Cortex samples and the contents of kukoamines A and B

Batch no.	Species	Origin	Collection year	Content of KA^a^ (mg/g)	Content of KB^a^ (mg/g)
LC 1	*L. chinense*	Jiangxi	2011	9.65 ± 0.26	5.03 ± 0.13
LC 2	*L. chinense*	Jiangxi	2011	4.38 ± 0.27	13.27 ± 0.04
LC 3	*L. chinense*	Jiangxi	2011	3.33 ± 0.18	13.50 ± 0.18
LC 4	*L. chinense*	Jiangxi	2011	1.82 ± 0.13	3.39 ± 0.22
LC 5	*L. chinense*	Gansu	2011	3.83 ± 0.03	13.29 ± 0.14
LC 6	*L. chinense*	Shangxi	2011	6.34 ± 0.11	22.08 ± 0.11
LC 7	*L. chinense*	Neimenggu	2011	1.13 ± 0.02	3.49 ± 0.02
LC 8	*L. chinense*	Neimenggu	2011	4.87 ± 0.04	14.27 ± 0.27
LC 9	*L. barbarum*	Ningxia	2011	1.86 ± 0.09	6.80 ± 0.18
LC 10	*L. barbarum*	Ningxia	2011	1.93 ± 0.02	6.65 ± 0.01
LC 11	*L. barbarum*	Ningxia	2011	4.06 ± 0.23	16.88 ± 0.38
LC 12	*L. barbarum*	Ningxia	2011	1.47 ± 0.01	4.76 ± 0.23
LC 13	*L. barbarum*	Ningxia	2011	3.96 ± 0.29	19.02 ± 0.11
LC 14	*L. chinense*	Hong Kong	2011	1.29 ± 0.03	3.47 ± 0.15
LC 15	*L. chinense*	Hong Kong	2012	4.25 ± 0.04	18.45 ± 0.60
LC 16	*L. chinense*	Hong Kong	2012	1.75 ± 0.02	7.60 ± 0.02

^a^The content was converted to the dry basis. The experiments for each sample were performed in triplicate (n = 3), and the results are presented as the mean ± standard deviation

LyC sliced samples, with the commercial names Hong Digupi (RLyC) and Bai Digupi (WLyC), were provided by herb stores from the Hehuachi herbal market in Chengdu (Sichuan Province, China). These samples had unclear sources, and were classified based on traditional experience. LyC extracts (Nos. 01–03) were bought from herbal extract companies in Sichuan, Shanxi, and Hubei. These extracts were claimed to be extracted from LyC.

### Chromatographic conditions

The HPLC analysis was performed on an Agilent 1260 system (Agilent, USA) equipped with an on-line degasser (G1322A; Agilent, USA), a binary bump (G1312C; Agilent, USA), an autosampler (G1329B; Agilent, USA), a column oven (G1316A; Agilent, USA), and a diode array detector (G1315D; Agilent, USA). A Zorbax C18 SB-AQ column (250 mm × 4.6 mm i.d., 5 μm; Agilent, USA) was used for the separation. A mobile phase consisting of 0.1% TFA (A) and acetonitrile (B) was applied for the separation with the following gradient program: 0–15 min, 12–16% B; 15–35 min, 16–22% B. The flow rate was 1.0 mL/min and the column oven was set at 40 °C. The detection wavelength was 280 nm.

### Sample preparation

Each LyC sample was cut into small pieces and pulverized into a powder (passed through a 200-mesh sieve). The herbal powder (0.5 g) was accurately weighed. The extraction solvent was a 50% methanol aqueous solution containing 0.5% acetic acid (v/v). After addition of 20 mL of the extraction solvent, the mixture was sonicated in an ultrasonic bath (100 W, AC-120H; MRC, Germany) for 30 min and then centrifuged at 2880×*g* for 10 min (Allegra X-15R; Beckman Coulter, USA). The supernatant was transferred into a 50-mL volumetric flask and the pellet was re-extracted with another 20 mL of extraction solvent for 30 min. After centrifugation, the extracted solution was transferred to the same volumetric flask. Next, 10 mL of extraction solvent was used to wash the tube, and the washing solution was also added to the volumetric flask and filled to the mark. The prepared sample solution was filtered through a 0.22-μm pore-size filter before injection.

### Method validation

Calibration curves for KA and KB were constructed by plotting the peak area versus the standard concentration. The calibration curves ranged from 3.91–250.00 mg/L for KA and 4.12–263.50 mg/L for KB. The method precision was measured by the relative standard deviation (RSD; %) of the peak areas determined for six successive injections of the standard solution. The repeatability test was measured by injection of five replicate samples for the proposed sample preparation and determination methods. The repeatability was calculated by the RSD of the contents obtained from the five replicate samples [17]. The sample recovery was assessed by adding known amounts of individual standards into an accurately weighted sample. Three concentrations were investigated and triplicate measurements were performed for each concentration. Recovery was calculated with the following equation [16]:

$${\text{Recovery}}\,(\% ) = 100 \times ({\text{amount found}} - {\text{original amount}})/{\text{amount}}\,{\text{spiked}}$$

The method detection limit (MDL) and limit of quantitation (LOQ) were determined according to the guidelines in Validation of Analytical Procedures: Methodology (Q2B) [18].

### Statistical analysis

The contents of samples were presented as the mean ± standard deviation. The proposed content limit for each analyte was calculated according to the contents from multiple batches with consideration of the standard uncertainty of the precision, bias, purity of reference substance, and water content (the calculation equation is shown in Additional file 1). The calculation was based on the ISO “Guide to the Expression of Uncertainty in Measurement” [19], EURACHEM/CITAC document “Quantifying Uncertainty in Analytical Measurement” [20], and LGC document “Development and Harmonization of Measurement Uncertainty Principles” [21]. The mean differences for KA and KB in the two species of *L. chinense* and *L. barbarum* were evaluated by analysis of variance (ANOVA). If the *P* value was less than 0.05, a follow-up post hoc test was conducted.

## Results and discussion

### Establishment of HPLC conditions for determination of kukoamines

#### Selection of column, mobile phase, and detection wavelength

KA and KB are water-soluble spermine alkaloids with multiple amino and amide groups [22, 23] that cause peak tailing on the C_18_ packing material. Three kinds of C_18_ columns with special coatings on the packing surface, i.e., Zorbax C_18_ BDS, Zorbax C_18_ Extended, and Zorbax C_18_ SB-AQ (all from Agilent, USA), were compared for the analyte retention behaviors. The kukoamines showed better retention on the Zorbax C_18_ SB-AQ column, owing to the better affinity of the extremely water-soluble analytes for the hydrophilic packing surface.

The influence of acids on the separation was investigated (Table 2). Within the pH range of 2.0–2.5, TFA was superior to phosphoric acid and formic acid in obtaining satisfactory peak resolution and symmetry. Furthermore, the peak sequence of KA and KB observed with TFA was the opposite to that observed with the other two acids. This difference was caused by the formation of ion pairs [24], which greatly altered the interaction between the analytes and the packing surface. Owing to the better separation performance, TFA was chosen as the acid for the mobile phase. The detection wavelength chosen for the kukoamines was 280 nm, based on the characteristic UV absorption of the *o*-dihydrocaffeoyl groups in the analytes.

**Table 2:** Influence of acids on chromatographic behaviors of kukoamines

Aqueous phase^a^ (v/v)	Analytes	Retention time (min)	Plates^b^	R^c^	T^d^
Water	KA	15.3	126	0.8	2.30
	KB	20.9	52		4.40
0.2% Formic acid (v:v) pH 2.5	KA	34.6	4557	1.0	3.02
	KB	37.8	4632		3.06
0.1% Phosphate acid (v:v) pH 2.0	KA	35.1	3954	0.7	2.01
	KB	37.9	3249		2.52
0.1% Trifluoroacetic acid (v:v) pH 2.0	KA	47.3	10,091	2.6	1.10
	KB	37.1	10,887		1.12

^a^The exp eriment was conducted on an Agilent Zorbax C_18_ SB-AQ column (250 mm × 4.6 mm i.d., 5 μm) with fixed proportions of acetonitrile and aqueous phase (16:84). The aqueous phase was investigated with different acids

^b^The number of theoretical plates was calculated as N = 5.54 (t_R_/W_h/2_)^2^, where t_R_ is the retention time and W_h/2_ is the peak width at half height

^c^The resolution R refers to the resolution between KA and KB. It was calculated as R = 2 (t_A_ − t_B_)/(W_A_ + W_B_), where t_A_ and t_B_ are the retention times and W_A_ and W_B_ are the peak widths

^d^The tailing factor T was calculated as T = W_0.05h_/2f, where W_0.05h_ is the peak width at 5% peak height and f is the width start point at 5% peak height to the time of the maximum point

### Influence of pH on the stability of the kukoamines

**Fig. 2:**
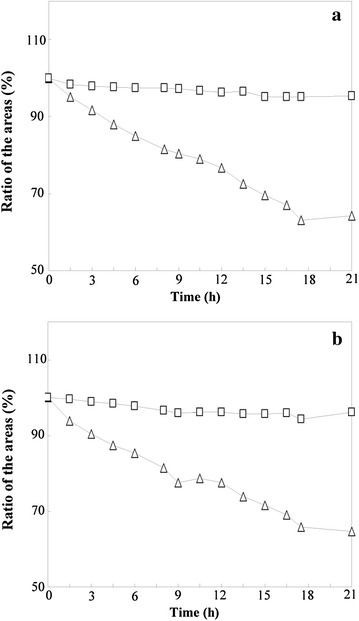
Kukoamines A (**a**) and B (**b**) are stable in sample solution at pH 2.5 (*open square*), but not at pH 7.0 (*triangle*)

A stability test for the analytes was performed, and the results are shown in Fig. 2. KA and KB were not stable in neutral solution (pH = 7), as they were found to decrease by about 30% after exposure in air for 21 h. This change might arise through oxidation of the *o*-dihydrocaffeoyl moieties into quinones [25]. In contrast, it was found that when the pH was adjusted to 2.5–3 by acetic acid, KA and KB remained constant within the investigated period. This protective effect can be explained by the formation of hydrogen bonds between the phenolic hydroxyl groups and the solvent molecules in the acidic solution, which greatly protected the phenolic hydroxyl groups from oxidization [26]. Therefore, an acidic solution (0.5% acetic acid aqueous solution, v/v) was preferred for both the standard and sample solutions.

### Optimization of kukoamine extraction procedures

**Fig. 3:**
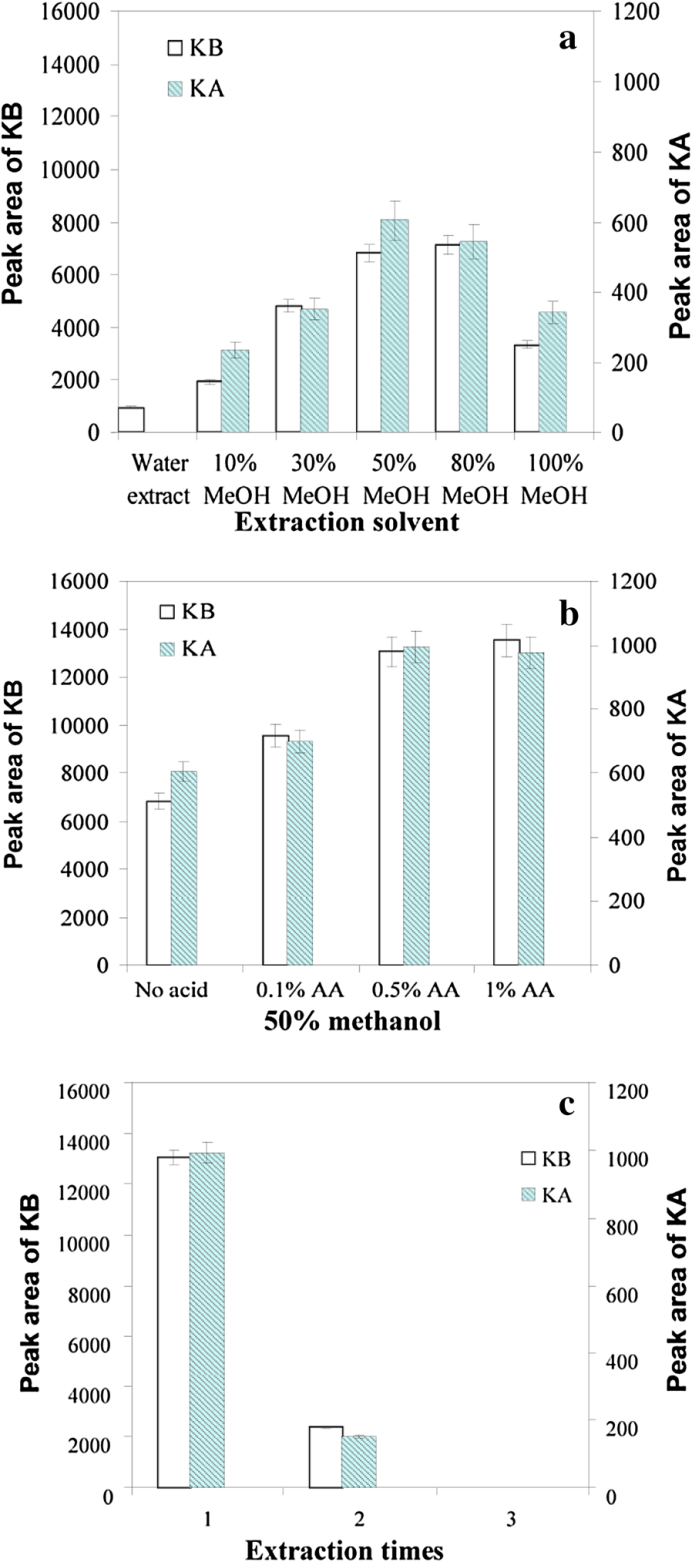
Influence of methanol concentration (**a**), acetic acid concentration (**b**), and number of extraction rounds (**c**) on the extraction efficiency of kukoamines in LyC. The experiments for each condition were performed in triplicate (n = 3) and the results are presented as the mean ± standard deviation

Key factors influencing the extraction efficiency, specifically the extraction solvent and number of extraction rounds, were optimized to completely extract the analytes for analysis. The 50 and 80% methanol solutions (v/v) showed similar performances, which were much higher than those for the other concentrations (Fig. 3a). The optimal concentration of methanol for extraction was found to be 50% (v/v) from the aspect of eco-friendliness. Figure 3b shows the effects of acids on the extraction efficiency. The acidic solution was about onefold more potent than the neutral solution for kukoamine extraction. Therefore, 0.5% acetic acid (v/v) was adopted, because it facilitated the release of alkaloids from the powder to the solvent. The influence of the number of extraction rounds was also examined (Fig. 3c). When samples were sonicated twice, almost all of the kukoamines were completely extracted. Therefore, two extractions were chosen for our extraction procedure.

### Method validation

The established method was validated for its detection limit (MDL), quantification limit (LOQ), linearity, precision, reproducibility, and recovery. As shown in Table 3, the high correlation coefficient (r^2^ = 0.9999; *P* < 0.05) indicated good linearity between the concentrations of kukoamines and their peak areas. The MDL and LOQ were derived from the responses of the analytes to UV, and were found to be 1.76 and 8.78 μg/mL for kukoamine A, and 1.86 and 9.29 μg/mL for kukoamine B, respectively. The RSD (%) in the recovery, precision, and reproducibility tests were all less than 2%. The good recovery obtained further suggested that the method was accurate and reliable.

**Table 3:** Linearity, precision, repeatability, recovery, MDL, and LOQ of the established method

Parameter	Kukoamine A	Kukoamine B
Linearity (12 points)	3.91–250.00 mg/L; r^2^ = 0.9999^a^	4.12–263.50 mg/L; r^2^ = 0.9999^a^
Precision (RSD)^b^, %	1.29	0.57
Repeatability (RSD)^c^, %	1.81	0.92
Mean recovery (n = 5)^d^	95.89% 90.03–102.30%	100.32% 98.49–101.67%
MDL (µg/mL)	1.76	1.86
LOQ (µg/mL)	8.78	9.29

*MDL* method detection limit, *LOQ* limit of quantification

^a^r^2^, square of correlation coefficient for the linear regression (*P* < 0.05)

^b^Precision was tested based on six injections of a standard solution with 62.50 µg/mL kukoamine A and 131.75 µg/mL kukoamine B. RSD (%) = 100 × SD/mean

^c^Repeatability was tested on five replicate samples

^d^Recovery (%) = 100 × (amount found − original amount)/amount spiked. The results were presented as the mean recovery and recovery range. The amounts of spiked components for the recovery test were 10.4, 20.8, and 41.6 mg for KB, and 1.5, 3.0, and 4.5 mg for KA

### Quantification results and proposed contents

**Fig. 4:**
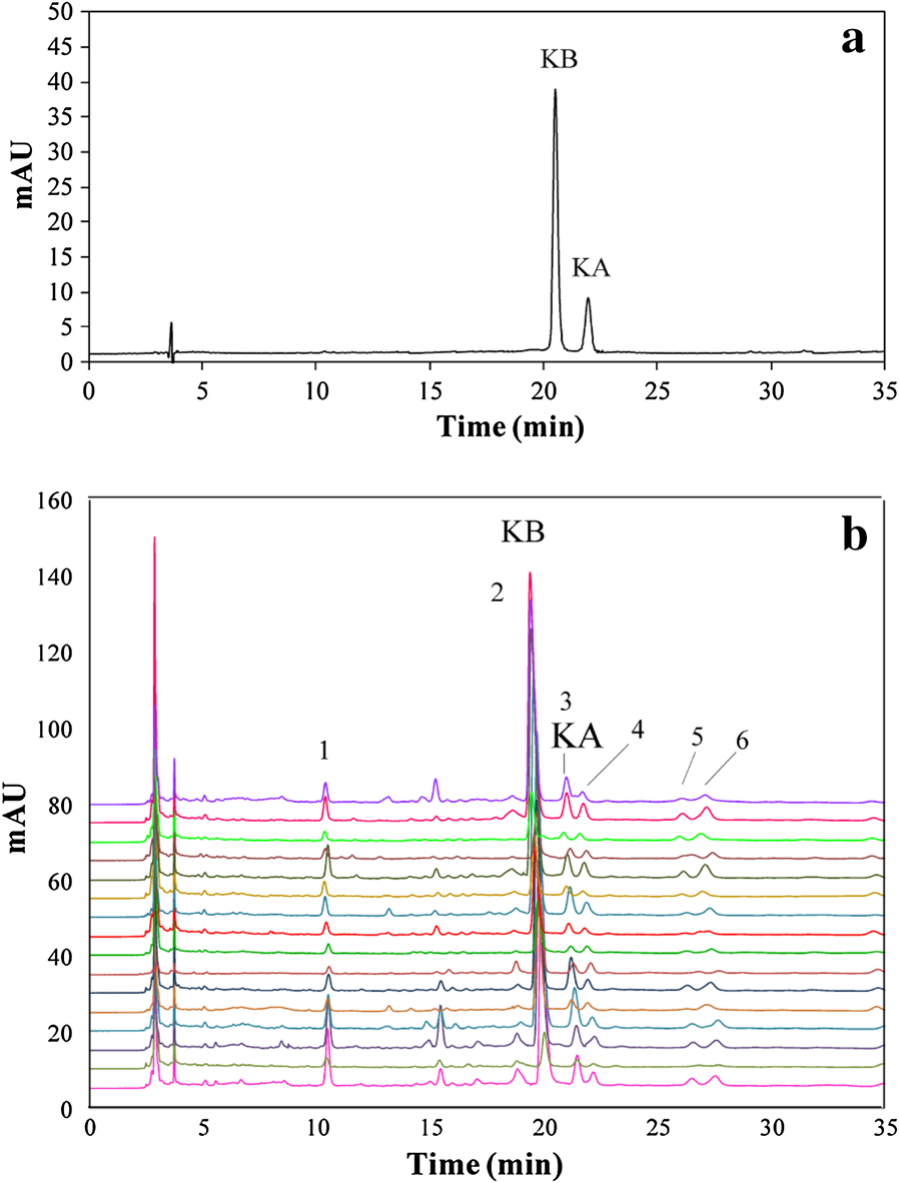
HPLC chromatogram of mixed standards (**a**) and fingerprint profiles of LyC (**b**) from multiple batches. The numbers 1–6 in **b** indicate the common fingerprinting peaks for identification

The validated method was applied to investigation of the LyC samples from different locations, which covered most of the production regions in China. The post-harvest treatment of the herb strictly followed the procedures described in the professional compendiums to ensure quality consistency [4, 27]. A typical chromatogram of LyC and the overlay chromatograms for multiple batches are shown in Fig. 4a, b. The chromatographic patterns of all samples, regardless of whether they were *L. barbarum* or *L. chinense*, were highly consistent, although the abundance of some peaks varied remarkably. With the proposed chromatographic methods, the common peaks (1–6) in Fig. 4b could be used as the fingerprinting features for LyC authentication. The contents of KA and KB in the investigated LyC samples were in the ranges of 1.13–9.65 and 3.39–22.08 mg/g, respectively (Table 1). In general, KB was more abundant than KA in the LyC samples.

The LyC samples from *L. chinense* and *L. barbarum* did not differ significantly from each other in their kukoamine contents when compared by one-way ANOVA (Additional file 1). These findings demonstrated that both plants can produce LyC with equivalent quality.

Geography, soil, and climate can cause fluctuations in herbal constituents [28–30]. Nevertheless, the secondary metabolites in LyC, KA and KB, typically fitted a normal distribution model [19–21]. Therefore, we estimated the content ranges of KA and KB for the LyC population using data from the collected representative samples by statistical inference [19–21] (Additional file 2). At the 95% confidence level, the calculated content limits were 1.45 mg/g for KA and 4.72 mg/g for KB (Table 4). These content limits could be used as reference values to judge the quality of LyC samples (Table 4).

**Table 4:** Mean contents and proposed content limits for kukoamines A and B in Lycii Cortex

Parameters	KA	KB
Ca^a^	3.49	10.75
SD^b^	2.51	6.31
RSD^c^	71.91	58.73
n^d^	16	
t_critical_^e^	2.131	
*uc* (C)^f^	0.46	2.67
Calculated limit^g^	1.45	4.72
Outlier^h^	0	0
Failing rate (%)^i^	12.50	18.75

^a^Ca, average content of klkukoamines in samples (mg/g)

^b^SD, standard deviation of the contents in all samples

^c^RSD (%) = 100 × SD/mean

^d^n, sample size

^e^t_critical_, critical value for a 95% confidence interval when the sample size was 16

^f^ *uc* (C), combined standard uncertainty of the kukoamine B content in the sample (mg/g). The calculation is shown in the supplementary data section

^g^Calculated limit for kukoamines in the sample. The calculation is shown in the supplementary data section

^h^Number of outlier samples. The data are referenced to Table 1

^i^Percentage of samples with kukoamine contents outside the calculated limits

### Application of the HPLC method for quality control of LyC

**Fig. 5:**
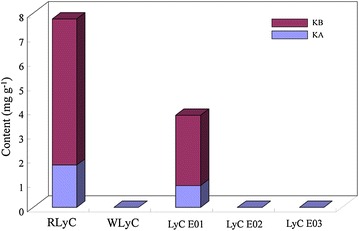
Quality assessment of LyC sliced samples and extracts from markets. *RLyC* Hong Digupi, *WLyC* Bai Digupi, *LyC E 01–03* LyC extracts from manufacturers 01–03

Five batches of unauthenticated samples, which were claimed to be LyC sliced pieces and extracts, were tested by the established method and then judged by the calculated quality criteria. As shown in Fig. 5, only the crude material called “Hong Digupi” and the extract powder from Sichuan presented the proper amounts of kukoamines, while other samples did not contain any kukoamines. The “Bai Digupi” on the market was actually not from the root bark of either *L. chinense* or *L. barbarum*. The lack of kukoamines in the investigated LyC extract powder might be caused by an inappropriate extraction method that damaged the major bioactive compounds, or extraction from the wrong source plants.

Compared with the conventional morphological identification, the proposed HPLC method for monitoring the characteristic constituents KA and KB provides a simple and universal way to measure the quality of LyC samples. The method and calculated content limits presented in this article could support amendments to the quality standards for LyC in official compendiums.

## Conclusion

The developed method could be applied for quality inspection of LyC in the food and pharmaceutical manufacturing industries.

## Additional files

**Additional file 1: Table S1.** Comparison of LyCs from *L. chinensis* and *L. barbarum* in terms of kukoamines A and B using ANOVA test.

**Additional file 2.** Equations for calculation of the content limits of kukoamines.

## Abbreviations

LyC: Lycii Cortex
KA: kukoamine A
KB: kukoamine B
HPLC: high performance liquid chromatography
LOQ: limit of quantification
MDL: method detection limit
RSD: relative standard deviation
TFA: trifluoroacetic acid

## References

[1] Ministry of Health of RP China. Document for further management of materials for manufacture functional food. 2002. http://www.moh.gov.cn/zhuzhan/wsbmgz/201304/e33435ce0d894051b15490aa3219cdc4.shtml.

[2] Yao X, Peng Y, Xu LJ (2011). Phytochemical and biological studies of Lycium medicinal plants. Chem Biodivers.

[3] Potterat O (2010). Goji (*Lycium barbarum* and *L. chinese*): phytochemistry, pharmacology and safety in the perspective of traditional uses and recent popularity. Planta Med.

[4] Committee of National Pharmacopoeia (2010). Pharmacopoeia of the People’s Republic of China.

[5] Ministry of Health (2012). Labor and welfare: The Japanese pharmacopoeia.

[6] Korea Food and Drug Administration (2007). The Korean herbal pharmacopoeia.

[7] Liang XM, Jin Y, Wang YP (2009). Qualitative and quantitative analysis in quality control of traditional Chinese medicines. J Chromatogr A.

[8] Lau AJ, Woo SO, Koh HL (2003). Analysis of saponins in raw and steamed *Panax notoginseng* using high-performance liquid chromatography with diode array detection. J Chromatogr A.

[9] Shi Z, He J, Yao T (2005). Simultaneous determination of cryptotanshinnone, tanshinone I and tanshinone IIA in traditional Chinese medicinal preparations containing Radix salvia miltiorrhiza by HPLC. J Pharm Biomed Anal.

[10] Wu HF, Guo J, Chen SL (2012). Recent developments in qualitative and quantitative analysis of photochemical constituents and their metabolites using liquid chromatography-mass spectrometry. J Pharm Biomed Anal.

[11] Chu QC, Fu L, Lin M (2005). Study on bioactive ingredients in Cortex Lycii by capillary zone electrophoresis with amperometric detection. Chin J Anal Chem.

[12] Li K, Chen XH, Bi KS (2005). Determination of vanillic acid in Cortex Lycii by RP-HPLC. Chin Tradit Herbal Drugs.

[13] Li K, Li Q, Yuan JS (2005). Determination of scopoletin in Cortex Lycii by RP-HPLC. Chin Pharm J.

[14] Li YY, Di R, Baibado JT (2014). Identification of kukoamines as the novel markers for quality assessment of Lycii Cortex. Food Res Int.

[15] Zhang JX, Guan SH, Yang M (2013). Simultaneous determination of 24 constituents in Cortex Lycii using high-performance liquid chromatography-triple quadrupole mass spectrometry. J Pharm Biomed Anal.

[16] Cheung HY, Li YY, Di R, Process for isolating kukoamine. USP 9012687B2.

[17] US Environmental Protection Agency. Technique support document for the assessment of detection and quantitation approaches. EPA-821-R-03-005, Washington D.C. 2003.

[18] ICH, H. T. G. Validation of Analytical Procedure: Methodology (Q2B). International conference on harmonization. USA; 1997.

[19] International Organization for the Standardization: ISO/IEC Guide 98-3 uncertainty of measurement—part 3: guide to the expression of uncertainty in measurement (GUM: 1995). Geneva; 2008.

[20] Ellison SLR, Roesslein M, Williams A. EURACHEM/CITAC Guide: quantifying uncertainty in analytical measurement, 2nd ed. 2000. http://www.vtt.fi/ket/eurachem/quam2.pdf.

[21] Barwick VJ, Ellison SLR. VAM Project 3.2.1 Development and Harmonisation of Measurement Uncertainty Principles Part (d): Protocol for Uncertainty Evaluation from Validation Data, Version 5.1, LGC (Teddington) Limited. 2000. http://blpd.dss.go.th/knowledge_el/VAM_uncertainty-0452.pdf.

[22] Parr A, Mellon FA, Colquhoun IJ (2005). Dihydrocaffeoyl polyamines (kukoamine and allies) in potato (*Solanum tuberosum*) tuber detected during metabolite profiling. J Agr Food Chem.

[23] Rogoza LN, Salakhutdinov NF, Tolstikov GA (2005). Plant alkaloids of the polymethyleneamine series. Russ Chem Rev.

[24] Cai B, Li J (1999). Evaluation of trifluoroacetic acid as an ion-pair reagent in the separation of small ionizable molecules by reversed-phase liquid chromatography. Anal Chim Acta.

[25] Li YY, Wang H, Zhao C, Huang Y, Tang X, Cheung HY (2015). Inentification and characterization of kukoamine metabolites by multiple ion monitoring triggered enhanced product ion scan method with a triple-quadruple linear ion trap mass spectrometer. J Agric Food Chem.

[26] Sjödin M, Irebo T, Utas JE, Lind J, Merényi G, Akermark B, Hammarström L (2006). Kinetic effects of hydrogen bonds on proton-coupled electron transfer from phenols. J Am Chem Soc.

[27] Wagner H, Bauer R, Melchart D (2011). Chromatographic fingerprint analysis of herbal medicines: thin-layer and high performance liquid chromatography of Chinese drugs (vol 1).

[28] Yi LZ, Yuan DL, Liang YZ (2007). Quality control and discrimination of Percarpium Citri Reticulatae and Pericarpium Citri Reticulatae Viride based on high-performance liquid chromatographic fingerprints and multivariate statistical analysis. Anal Chim Acta.

[29] Liang YZ, Xie PS, Chan K (2004). Quality control of herbal medicines. J Chromatogr A.

[30] Zhao ZZ, Yuen JPS, Wu J (2006). A system study on confused species of Chinese Materia Medica in the Hong Kong market. Ann Acad Med Singapore.

