# Supplementary material for: Quality control of Lycium chinense and Lycium barbarum cortex (Digupi) by HPLC using kukoamines as markers
Source: Chin Med. 2017 Jan 9;12:4. doi: 10.1186/s13020-016-0121-x (PMC5223573; doi:10.1186/s13020-016-0121-x)
Supplement: Supplementary file 1 — Additional file 1: Table S1. Comparison of LyCs from L. chinensis and L. barbarum in terms of kukoamines A and B using ANOVA test. [file 13020_2016_121_MOESM1_ESM.docx]

**Table S1 Comparison of LyCs from *L. chinensis* and *L. barbarum* in terms of kukoamines A and B using ANOVA test**

Table 1S. ANOVA test for the mean difference of kukoamines A and B in the two species.

| Source of Variation | Compounds | SS ^a^ | Df ^b^ | MS ^c^ | F ^d^ | F crit^e^ |
| --- | --- | --- | --- | --- | --- | --- |
| Between Groups | KA | 5.1186 | 1 | 5.1186 | 1.0199 | 4.6001 |
|  | KB | 0.0395 |  | 0.0395 | 0.0009 |  |
| Within Groups | KA | 70.2601 | 14 | 5.0186 |  |  |
|  | KB | 597.5173 |  | 42.6798 |  |  |
| Total | KA | 75.3787 | 15 |  |  |  |
|  | KB | 597.5568 |  |  |  |  |

^a^ Sum of square

^b^ Degree of freedom

^c^ Mean square

^d^ Calculated F value

^e^ Critical F at 95% confidence interval.
