# Supplementary material for: Quality control of Lycium chinense and Lycium barbarum cortex (Digupi) by HPLC using kukoamines as markers
Source: Chin Med. 2017 Jan 9;12:4. doi: 10.1186/s13020-016-0121-x (PMC5223573; doi:10.1186/s13020-016-0121-x)
Supplement: Supplementary file 2 — Additional file 2. Equations for calculation of the content limits of kukoamines. [file 13020_2016_121_MOESM2_ESM.docx]

**Equations for calculation of the content limits of kukoamines**

In this study, the content limits of kukoamines were estimated with regard to the amount of analytes from multiple batches of samples concerning the uncertainties caused by multiple factors which may induce variation on determination results. Normally, for herbal quality analysis, those factors includes water content, standard purity, run-to-run precision and bias. In general, the combined uncertainty was calculated with equation (1) by the square root of the total variance obtained from each of the uncertainty components, and the content limit was estimated with equation (2) (International Organization for the Standardization, 2008; Ellision et al., 2000; Barwick and Ellison, 2000):

u_c_(C_a_)/C_a_ = (1)

where Ca is the content of kukoamine A (or B) in the sample (mg kg^-1^); u_c_(C_a_) is the combined standard uncertainty of contents of kukoamine A (or B) in sample (mg kg^-1^); *u* (Pr_a_), *u* (Rec_a_) and *u* (C*w*) are the standard uncertainty of the precision, bias, purity and water content.

L = (2)

cacuated from equation 1ytvestigated analytested om equation where n = the number of samples; t(n-1) is the t value at 95% level of significant, which is 2.131 for n=16; *s.d*. is the standard deviation of contents in samples with regard to the investigated analytes; *uc*(C) is the extended uncertainty which is 2-fold of the u_c_(C_a_) calculated from equation 1.
